# The Relationship Between Soil-Transmitted Helminth Infections and Environmental Factors in Puerto Iguazú, Argentina: Cross-Sectional Study

**DOI:** 10.2196/41568

**Published:** 2023-11-07

**Authors:** Ernesto Candela, Carolina Goizueta, Leonardo Sandon, Carla Muñoz-Antoli, Maria Victoria Periago

**Affiliations:** 1 Department of Pharmacy Pharmaceutical Technology and Parasitology Universitat de València Burjassot Spain; 2 Mundo Sano Foundation Buenos Aires Argentina; 3 National Scientific and Technical Research Council (CONICET) Buenos Aires Argentina

**Keywords:** soil-transmitted helminths, hookworm, prevalence, intensity: distribution: Iguazú, Argentina

## Abstract

**Background:**

Soil-transmitted helminths (STHs) are widely distributed throughout the world. Various factors, including the environment, socioeconomic characteristics, and access to water and sanitation, play an important role in the spread and persistence of these parasites within communities. They, in turn, affect the growth and development of members of the community, especially children. Studies in the northern provinces of Argentina have shown variable prevalence of STHs, but the factors associated with their presence have not been completely elucidated.

**Objective:**

This cross-sectional study aimed to identify the socioeconomic and environmental factors related to STH infection in indigenous villages located in Puerto Iguazú (Misiones), Argentina.

**Methods:**

Between 2018 and 2019, stool samples were collected from individuals ≥1 year residing in 3 villages: Mini-Marangatú, Yriapú, and Fortín Mbororé. Standard parasitological methods were used to determine STH prevalence. Standardized questionnaires were used to assess participants’ habits, customs, and household characteristics, and environmental data were obtained through satellite imagery. Multilinear regression with Akaike information criterion stepwise variables was used to explore relevant associations.

**Results:**

A total of 342 individuals from the 3 villages participated in this study. The prevalence of STHs varied across villages: 89.6% (43/48), in Mini-Marangatú, 80.8% (101/125) in Yriapú, and 68.5% (115/169) in Fortín Mbororé. Notably, there was a significant difference in hookworm infection among the villages (*P*=.02). The analysis highlighted the significant influence of specific environmental factors on STH presence and spatial distribution, particularly in relation to hookworm infection. Vegetation patterns represented by the Vegetation Heterogeneity Index, created ad hoc for this study, emerged as a critical factor, with 2 significant predictors related to it (*P*=.002 and *P*=.004) alongside impervious surface density with a significant predictor *(P*<.001). The multilinear regression model yielded a high *F* test score (*F*_108_=4.75, *P*<.001), indicating a strong fit (*R*^2^=0.5465). Furthermore, socioeconomic factors, including walking barefoot in houses with dirt floors and overcrowding, were significantly correlated with hookworm infection intensity (*P*<.001 and *P*=.001, respectively). We also used the multilinear regression model to calculate hookworm infection intensity (*F*_110_=21.15, *P*<*.*001; *R*^2^=0.4971).

**Conclusions:**

Our study underscores the complexity of STH transmission, as villages with similar living conditions and environmental characteristics displayed varied STH prevalence and spatial distribution. Specific environmental factors, such as vegetation pattern and impervious surface density, played major roles in STH presence, demonstrating the crucial relationship between environmental factors and hookworm infection distribution. Moreover, our findings emphasize the significant influence of socioeconomic factors on hookworm infection intensity. By gaining insights into this complex interplay, our research contributes to a better understanding of STH transmission characteristics, thereby informing targeted public health interventions for effective control.

## Introduction

Soil-transmitted helminth (STH) infections are the most prevalent among the neglected tropical diseases (NTDs) worldwide, affecting over 1.5 billion people as of 2023 [1]. These diverse diseases have an enormous impact on individuals, families, and communities in low- and middle-income countries, and they constitute a serious obstacle to socioeconomic development and quality of life, leading to loss of productivity and exacerbating poverty [2]. NTDs are mainly prevalent in tropical and subtropical areas, where they mostly affect impoverished communities and disproportionately affect women and children [2,3]. STHs are intestinal parasites (IPs), with the most common species affecting humans being *Ascaris lumbricoides, Trichuris trichiura,* and the hookworms *Necator americanus* and *Ancylostoma duodenale* [4]. Although *Strongyloides stercoralis* is a common STH in Latin America and the Caribbean, it is not included in this group due to its complex life cycle and specific characteristics for diagnosis, quantification, and treatment [5,6].

Previous studies have shown the involvement of different factors in the transmission of STHs, including socioeconomic factors, such as the Human Development Index; nutritional and immunological factors; and environmental factors, such as the Normalized Difference Vegetation Index (NDVI) and the Enhanced Vegetation Index (EVI) [7-9]. All these factors exert an economic impact on the population and thus also play a role in the perpetuation of poverty [10,11].

Argentina has a heterogeneous prevalence of STHs (between 0% and 88.9%) throughout the country, and the northeast and northwest provinces of Misiones, Chaco, or Salta are identified as endemic [12-16], with varying rates of infection depending on socioeconomic status, sanitary and environmental conditions, and access to water [16-19]. Despite its high prevalence of STHs, Argentina does not currently have a deworming program as approved by the World Health Assembly (WHA) through resolution WHA54.19 [20].

The Misiones province is composed of 10,218 indigenous people from the Mbyá-Guaraní ethnic group distributed among 116 communities that live mostly in rural areas [21,22] under precarious and poor sanitary conditions, with high rates of malnutrition among children [17]. IPs are highly endemic in this area, especially among indigenous communities [23], with varying prevalence rates depending on age, hygiene habits, access to water and basic sanitation, and nutritional conditions, among others [16,17,24]. These factors can cause or aggravate malnutrition, leading to anemia and growth delays in children [18,25].

Geographic information system (GIS), remote sensing (RS), and digital elevation model (DEM) technologies provide information to identify and analyze determinants of disease distribution, thereby facilitating the development of risk prediction models in relation to environmental variables. This, in turn, aids the design of strategies to identify and prevent these infections. In this study, we collected parasitological and socioeconomic data from different Mbyá-Guaraní villages of Puerto Iguazú, Misiones, Argentina, as well as environmental variables, to determine the main factors associated with STH infection in this region.

## Methods

### Study Area

Puerto Iguazu is a city located in the province of Misiones in northeastern Argentina. It is naturally divided by the Paraná and Iguazú Rivers, which act as the physical borders between Argentina, Brazil, and Paraguay. According to the most recent national census data, the city has over 42,800 inhabitants [26].

**Figure 1:**
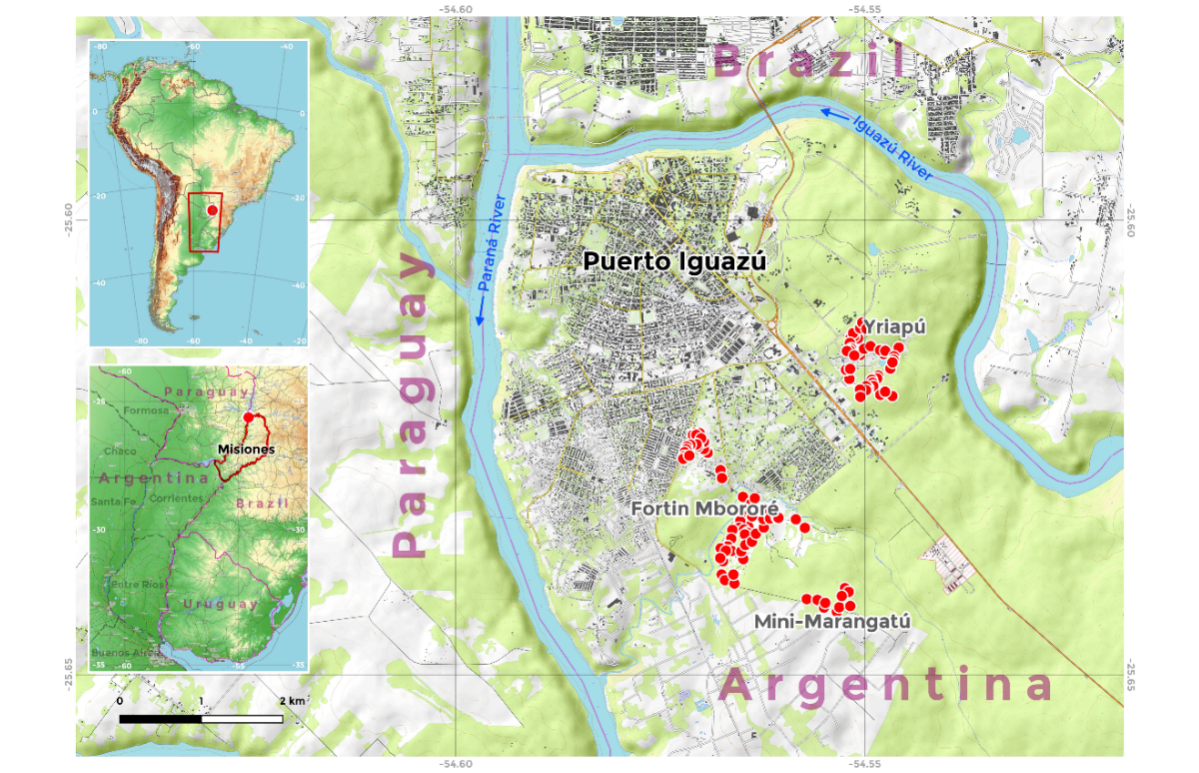
Map of the study area. Points represent each of the georeferenced households from the 3 villages included in the study, Fortín Mbororé, Mini-Marangatú, and Yriapú, which are adjacent to Puerto Iguazú, Misiones, Argentina. Map created using QGIS with overlay imagery from OpenTopoMap (CC-BY-SA) via the QuickMapServices plugin (version 0.19.29). Copyright belongs to OpenStreetMap contributors [28]. Map data are from August 27, 2021.

This study was carried out in the rural area of Puerto Iguazú, around the city´s periphery, where Mbyá Guaraní indigenous communities have settled into 3 villages: Fortín Mbororé, Mini-Marangatú, and Yriapú. Previous studies in this area have shown a high prevalence of IPs, specifically STHs [12,16,27]. Moreover, these villages are adequately sized to enable the enrollment of enough participants to explore the relationship between STH infection and its determinants, with 200 families in Fortín Mbororé, 35 in Mini-Marangatú, and 100 in Yriapú (Figure 1).

The family subsistence economy of the communities that participated in this study is from guided tours organized to visit the village, handcrafts, and social plans [12]. These communities share similar water and sanitation conditions and are homogeneous in their economic status, with low monthly incomes. Living conditions are characterized by a lack of water and sanitation [19]. Houses are scattered, surrounded by vegetation, and made of adobe bricks with unimproved roofs and dirt floors.

### Study Design

Stool samples were collected from participants from all 3 villages. The households were visited, georeferenced, and characterized using a questionnaire. Stool containers were provided, along with verbal instructions on how to collect the samples, and retrieved on the following day. The fresh samples were transported without a fixative in a refrigerated icebox and kept at 4 °C in the lab until analysis within 24 hours of collection. The inclusion criteria were based on age (participants had to be at least 1 year of age) and willingness to participate, evidenced through the informed consent process. Individuals who lived or worked for long periods of time outside the study area and those with conditions that impaired an understanding of the consent process were excluded from the study. Considering that the area is in the tropical forest biome, which involves deforestation of tropical and subtropical forests for agriculture, the extension of grasslands may affect the viability of STH eggs and larvae. Nevertheless, several publications have suggested that the forest mass is in the process of forest transition, where the recovery of natural systems such as forests is taking place [29].

### Ethics Approval

The institutional review board of the Ministry of Health of the Province of Misiones approved this study protocol and consent forms (171403/2018). Written consent was obtained from all the parents/guardians for children under 16 years of age. All individuals aged 16 years and older are considered adults in Argentina; therefore, written consent was obtained from them directly.

### Parasitological Analysis

The collected stool samples were analyzed using the Ritchie concentration technique, Baermann concentration, and Kato-Katz technique to measure the infection intensity of STH, as previously described [12]. The parasitological parameters used were the prevalence of IPs and the intensity of STH infections in eggs per gram of feces.

### Living Conditions and Environmental Characterization

Habits, customs, and household characteristics were analyzed at the household level using GIS. The variables, which were collected using a standardized questionnaire, are summarized in Table 1.

**Table 1:** Variables analyzed at individual, household, and village levels.

Variables		Description	Results
Education		Education level of household inhabitants	From none to university level
Income		Source of income	Formal employment, public sector, tourism, animal farming, crafts, and social plans, among others
Animal farming		Presence of pets or animal farming	Dogs, cats, chickens, pigs, ducks, and other
Health coverage		Type of health insurance	Public system, private health insurance, and prepaid health insurance, among others
**Household characteristics**			
	Roof	Type of material	Wood, branches, adobe, and metal sheets
	Wall	Type of material	Wood, cement, dirt, bricks, and adobe
	Floor	Type of material	Wood, dirt floor, cement, and smoothed floor
	Electricity	Source of electricity	None, public network, generator, and other
	Water source	For human consumption, cooking, or washing	Borehole, tap water, well, and other
	Water treatment	Any type of treatment	Boiling, chemical treatment, and other
	Excreta disposal	Excretal disposal	Open defecation or latrine
	Cooking	Source of heat for cooking	Gas stove or oven, electric stove, wood stove, and other
	Garbage disposal	Type of disposal	Municipal system, burning, burying, and other
**Behavioral aspects**			
	Barefoot	Use of footwear	Yes or no
	Hand washing	Practice of handwashing before eating and after defecation	Yes or no

Environmental and geographical data were collected using RS together with DEM. From the RS, the Soil-Adjusted Vegetation Index (SAVI), Vegetation Heterogeneity Index (VHI), Enhanced Normalized Difference Impervious Surfaces Index (ENDISI), and Bare Soil Index (BSI) were obtained. From the DEM, the Topographic Position Index (TPI) and Topographic Wetness Index (TWI) were calculated. The characteristics and sources of these indices are detailed in Table 2 and Multimedia Appendix 1.

**Table 2:** Environmental and geographical indices used to determine their association with the presence of soil-transmitted helminths (STH) in villages from Puerto Iguazú, Misiones, Argentina.

Index	Source	Characteristics
Topographic Position Index [30]	30-m resolution DEM^a^ from the IGN^b^ [31]	This index measures the altitude of a point with respect to its surrounding area. Positive values indicate that the central point is located higher than its average surroundings (such as ridges and hilltops), negative values indicate a position lower than the average (valley and sinkholes), and a near-zero value indicates a flat or continuous slope. The index used is a multiscale mean of 100-, 200- and 500-meter radii.
Topographic Wetness Index [32]	30-m resolution DEM from IGN [31]	It identifies potential points of water accumulation (humidity) based on topographic elements. This index is used as a proxy for the measurement of soil humidity.
Soil-Adjusted Vegetation Index [33]	2019 mean annual surface reflectance from Sentinel 2 imagery [34], retrieved and processed with GEE^c^ [35]	This is a modification of the Normal Density Vegetation Index, which corrects for the brightness of the soil when vegetation is scarce. It is used to estimate the quantity, quality, and development of vegetation through RSd.
Vegetation Heterogeneity Index	2019 mean annual surface reflectance from Sentinel 2 imagery [34], retrieved and processed with GEE [35]	The index is used to identify areas of more or less vegetation with respect to their surroundings (80 m radius). The SD of these values was also mapped to show the heterogeneity of the vegetation in the study area. Low values indicate a subtle variation, while high levels show abrupt changes in the vegetation around each household.
Enhanced Normalized Difference Impervious Surfaces Index [36]	2019 mean annual surface reflectance from Sentinel 2 imagery [34], retrieved and processed with GEE [35]	It detects impervious surfaces (buildings, asphalted roads, etc). A threshold of 0.15 was applied to set a value of 1 for impervious areas and 0 for other surfaces. This binary map was used to get a map with the imperviousness index within a radius of 500 m for each household, with continuous values from 0 to 1.
Bare Soil Index [37]	2019 mean annual surface reflectance from Sentinel 2 imagery [34], retrieved and processed with GEE [35]	This index combines blue, red, and infrared bands to capture variations in the soil. Short infrared and red bands are used to quantify the mineral composition of the soil, while blue bands and infrared bands are used for vegetation cover. Values range between −1 and 1, with higher values indicating bare soil.

^a^DEM: digital elevation model.

^b^IGN: Instituto Geográfico Nacional (National Geographical Institute).

^c^GEE: Google Earth Engine.

^d^RS: remote sensing.

### Statistical Analyses

Data were analyzed using Stata 12 software (StataCorp) and RStudio (R Foundation for Statistical Computing). Measures were evaluated using proportion with 95% CIs and means with SDs. The chi-square test was used to compare significant associations between different variables. The age variable was categorized into 5 groups: group 1 (0-5 years), group 2 (6-10 years), group 3 (11-20 years), group 4 (21-40 years), and group 5 (>40 years).

To determine the spatial distribution of STH infection in the study area, an algorithm based on the kernel density estimation (KDE) [38-40] technique was used to identify areas where infection was more prevalent than expected, assuming a homogenous distribution (null hypothesis), by calculating the difference from it using the SD as the unit of measurement. Therefore, positive or negative values indicated higher or lower values of infection than expected under the null hypothesis. With the main results and this same method (a quartic kernel shape and bandwidth of 200 m), the distribution of other variables was calculated: intensity of hookworm infection, households with dirt floors where inhabitants walked barefoot, and households with overcrowding. Variables that showed a significant correlation with the presence and intensity of hookworm infection were then used in a multiple linear regression analysis [41]. Predictors of infection were selected using a stepwise method that selected the best predictors using the Akaike information criterion. Values were considered significant at *P*<.05, with a 95% CI. The full statistical report is available in Multimedia Appendix 2.

## Results

### Study Population

A total of 342 individuals from the 3 villages participated in this study and provided stool samples: 169 (49.4%) individuals from Fortin Mbororé, 125 (44.5%) from Yriapú, and 48 (14%) from Mini-Marangatú. The population distribution in the 3 communities was 53.8% (91/168) men and 46.2% (78/169) women in Fortin Mbororé, 56.8% (71/125) men and 43.2% (54/125) women in Yriapú, and 50% (24/48) for both sexes in Mini-Marangatú. The mean age of participants was 21 (SD 17.9) years in Fortin Mbororé, 10.4 (SD 11.43) years in Yriapú, and 15 (SD 12.21) years in Mini-Marangatú.

### Prevalence of IPs

The overall prevalence of IPs in the 3 villages was 95.8% (46/48) in Mini-Marangatú, 95.2% (119/125) in Yriapú, and 91.1% (154/169) in Fortín Mbororé. Protozoan infection ranged from 87.5% (42/48) in Mini-Marangatú to 81.6% (102/125) in Yriapú, while helminth infections were highest in Mini-Marangatú (44/448, 91.7%), followed by Yriapú (104/125, 83.2%) and lower in Fortin Mbororé (126/169, 74.6%) (Table 3). The STH prevalence was 68.1% (115/169) in Fortín Mbororé, 80.8% (101/125) in Yriapú, and 89.6% (43/48) in Mini-Marangatú. Infection caused by *T. trichiura* was only detected in Mini-Marangatú village, with only 1 (2.1%) case, and no *A. lumbricoides* infections were detected in this village. The most prevalent STH was hookworm, reaching statistically different infection rates (χ^2^_2_=7.6, *P*=.02) between the 3 villages: Mini-Marangatú (42/48, 87.5%), Yriapú (92/125, 73.6%), and Fortín Mbororé (114/169, 67.5%). The descriptive characteristics of STH infections in the 3 villages are provided in Table 3.

**Table 3:** Descriptive characteristics and prevalence of intestinal parasites in individuals from Fortin Mbororé, Yriapú, and Mini-Marangatú.

Characteristics		Fortin Mbororé	Yriapú	Mini-Marangatú
Age (years), mean (SD)		21 (17.9)	10.4 (11.43)	15 (12.21)
Age range		1-87	1-54	1-49
**Gender, n (%)**				
	Female	78 (46.2)	54 (43.2)	24 (50)
	Male	91 (53.8)	71 (56.8)	24 (50)
**Prevalence of protozoans, n (%); range (95% CI)**		138 (81.7); 75-86.8	102 (81.6); 73.7-87.5	42 (87.5); 74.2-94.4
	*Entamoeba coli*	69 (40.8); 33.6-48.5	73 (58.4); 49.5-66.8	22 (45.8);32-60.4
	*Entamoeba complex*	18 (10.7); 6.8-16.3	12 (9.6);5.5-16.3	3 (6.3);1.9-18.3
	*Entamoeba hartmanni*	19 (11.2); 7.3-17	24 (19.2); 13.1-27.2	12 (25); 14.5-39.6
	*Endolimax nana*	34 (20.1); 14.7-26.9	13 (10.4); 6.1-17.2	10 (20.8); 11.3-35.2
	*Iodamoeba butschlii*	10 (5.9); 3.2-10.7	2 (1.6);0.4-6.3	1 (2.1); 0.3-14.2
	*Chilomastix mesnili*	6 (3.6); 1.6-7.7	16 (12.8); 7.9-20	6 (12.5); 5.6-25.8
	*Giardia intestinalis*	40 (23.7); 17.8-30.7	38 (30.4); 22.9-39.1	14 (29.2); 17.8-44
	*Blastocystis* spp.	93 (55); 47.4-62.4	56 (44.8); 36.2-53.7	32 (66.7); 51.8-78.8
**Prevalence of helminths, n (%); range (95% CI)**		126 (74.6); 67.4-80.6	104 (83.2); 75.5-88.8	44 (91.7); 79.2-96.9
	*Enterobius vermicularis*	3 (1.8); 0.6-5.4	5 (4); 1.7-9.4	—^a^
	*Hymenolepis nana*	28 (16.6); 11.6-23	36 (28.8); 21.5-37.5	6 (12.5); 5.6-25.8
	*Trichuris trichiura*	—	—	1 (2.1); 0.3-14.2
	*Ascaris lumbricoides*	3 (1.8); 0.6-5.4	21 (17.2); 11.4-25.1	—
	Hookworm	114 (67.5); 60-74.2	92 (73.6); 65.1-80.7	42 (87.5); 74.2-94.4
	*Strongyloides stercoralis*	11 (6.5); 3.6-11.4	44 (35.48); 27.5-44.4	14 (29.2); 17.8-44

^a^—: not available.

Hookworm infection was higher in the age groups ranging from 0 to 5 years and from 6 to 10 years in Yriapú and lower in Fortin Mbororé, especially in the western area located near the urban area of Puerto Iguazú. Moreover, statistical differences between age groups were observed within Fortin Mbororé (*χ^2^*_4_=27.9, *P*<.001) and Mini-Marangatú (*χ^2^*_4_=17.5, *P*=.002). Mixed infections with different STH species were also observed (Tables 4 and 5), together with the intensity of infection. *Trichuris trichiura* and *A. lumbricoides* were present mainly as light-intensity infections. On the other hand, heavy-intensity hookworm infections were detected in all 3 villages. The highest rate of individuals with heavy infections (22/42, 52.4%) were found in Mini-Marangatú, but no statistical differences were observed between the types of intensity.

**Table 4:** Intensity of soil-transmitted helminth (STH) infections in individuals from Fortin Mbororé, Yriapú, and Mini-Marangatú.

Infections	Fortin Mbororé, n (%)				Yriapú, n (%)				Mini-Marangatú, n (%)		
	Light	Moderate	Heavy	Light		Moderate	Heavy	Light		Moderate	Heavy
Hookworm	67 (58.8)	12 (10.5)	35 (30.7)	61 (66.3)		11 (12)	20 (21.7)	15 (35.7)		5 (11.9)	22 (52.4)
*A. lumbricoides*	2 (66.7)	—^a^	1 (33.3)	12 (57.1)		6 (28.6)	3 (14.3)	—		—	—
*T. trichiura*	—	—	—	—		—	—	1 (100)		—	—

^a^—: not available.

**Table 5:** Number of mixed soil-transmitted helminth (STH) infections in individuals from Fortin Mbororé, Yriapú, and Mini-Marangatú.

Mixed STH infections	Fortin Mbororé, n (%)	Yriapú, n (%)	Mini-Marangatú, n (%)
Hookworm/*S. stercoralis*	10 (76.9)	37 (56.9)	13 (92.9)
Hookworm/*A. lumbricoides*	3 (23.1)	19 (29.2)	—^a^
Hookworm/*S. stercoralis*/*A. lumbricoides*	—	9 (13.9)	—
Hookworm/*S. stercoralis*/*T. trichiura*	—	—	1 (7.1)

^a^—: not available.

### Living Conditions

Living conditions between the villages were usually similar. The average number of inhabitants per household was 5.3 for Fortin Mbororé, 5.1 for Yriapú, and 5.8 for Mini-Marangatú. Most households had a single room for sleeping; therefore, overcrowding was common. Generally, houses were made of wooden walls and dirt floors—90% (43/48) in Mini-Marangatú and 50% (61/121) in Yriapú. In the case of Fortin Mbororé, this figure was reduced to 35% (58/166) since 40% (66/166) of the households had cement floors. Practically the entire population, both children and adults from all 3 villages, walked barefoot. Although 22.9% (38/166) of households practiced open defecation, most had a latrine that consisted of a simple ground excavation. With respect to the source of drinking water, all the families in Mini-Marangatú obtained their water from boreholes, along with 75% (92/121) in Yriapú and 49% (81/166) in Fortin Mbororé. Family incomes were low and precarious, mostly coming from animal farming, crafts, or social plans. In the newer village of Mini-Marangatú, which branched off from Fortin Mbororé, 60% (99/166) of the families obtained their income from crafts.

Only the type of floor was observed to be associated with hookworm transmission, with a significantly higher prevalence of hookworm found in individuals from Yriapú village living in dirt floor houses (*χ^2^*_3_=8.8, *P*=.03). Lack of sanitation and hygiene, water source, the use of a latrine with simple ground excavation, and source of income were not related to a higher prevalence of hookworms. Walking barefoot and living in overcrowding conditions were significantly related to the intensity of hookworm infection (*P*<.001 and *P*=.003, respectively; *F*_110_=46.2, *P*<.001), indicating the significance of the model (*R*^2^=0.46).

### Environmental Characterization

**Figure 2:**
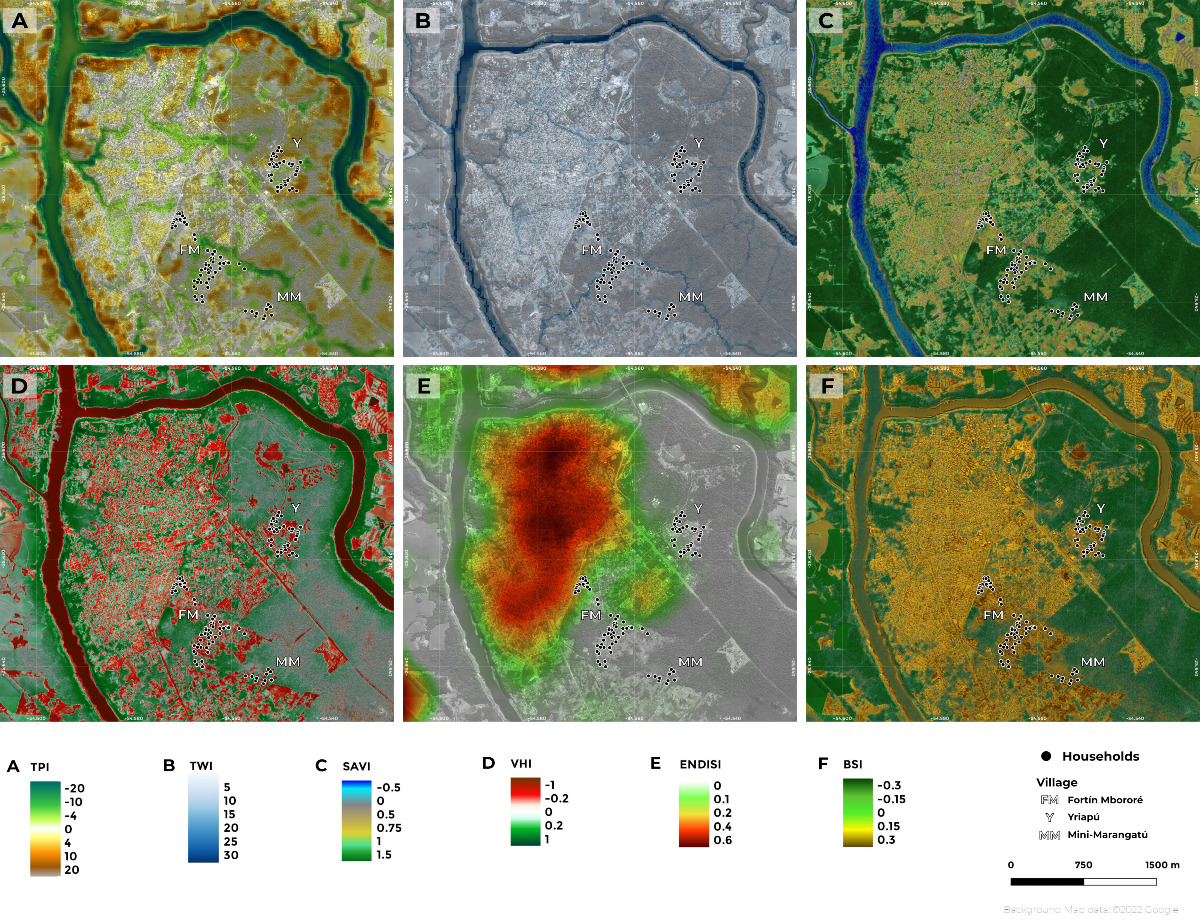
Distribution of the different indexes used in the study area from Puerto Iguazú, Misiones, Argentina. (A) Topographic Position Index (TPI). (B) Topographic Wetness Index (TWI). (C). Soil-Adjusted Vegetation Index (SAVI). (D) Vegetation Heterogeneity Index (VHI). (E) Enhanced Normalized Difference Impervious Surface Index (ENDISI). (F) Bare Soil Index (BSI). Map created using QGIS with background imagery from Google Maps via the QuickMapServices plugin (version 0.19.29). Copyright 2021 Google. Map data 2021 Google.

Figure 2 shows the distribution of the different environmental indices in the study area. TPI helps discriminate between areas that are depressed and those that have some prominence and thus are less prone to accumulating water, and TWI detects hydrological flow paths and thus proximity to streaming water and other bodies of water. The values of these 2 indices indicate that the study area was irregular with depressed areas and small hills (Figure 2A), with many water courses running through it, including the Mbocay stream (Figure 2B). Yriapú is located at the edge of a slightly depressed area, while Fortin Mbororé is divided into 2 parts by the Mbocay stream. Both sides of the village are on flat ground. Mini-Marangatú is located close to the crest of a small hill.

For the presence of vegetation, SAVI and VHI were used, with SAVI measuring vigor and VHI estimating the heterogeneity of the vegetation landscape. As depicted in Figures 2C and D, some differences in the distribution of the SAVI were observed between the villages. Western Fortin Mbororé had lower values than the central area of the village, while both Mini-Marangatú and Yriapú were surrounded by more vigorous vegetation (Figure 2C). Through the VHI, the difference between bare soil and the presence of vegetation was greatest in Mini-Marangatú and very small in Fortin Mbororé. The other 2 indices, ENDISI and BSI, were used to indicate the presence of bare soil (Figure 2E and 2F) but with different focuses: ENDISI on urbanized areas and BSI on natural soil. Again, Fortin Mbororé had a greater presence of bare soil around the houses, especially in the western area where households were located close to the urban city of Puerto Iguazú, whereas both Yriapú and Mini-Marangatú had patches of bare soil and vegetation.

### Spatial Distribution of STH

**Figure 3:**
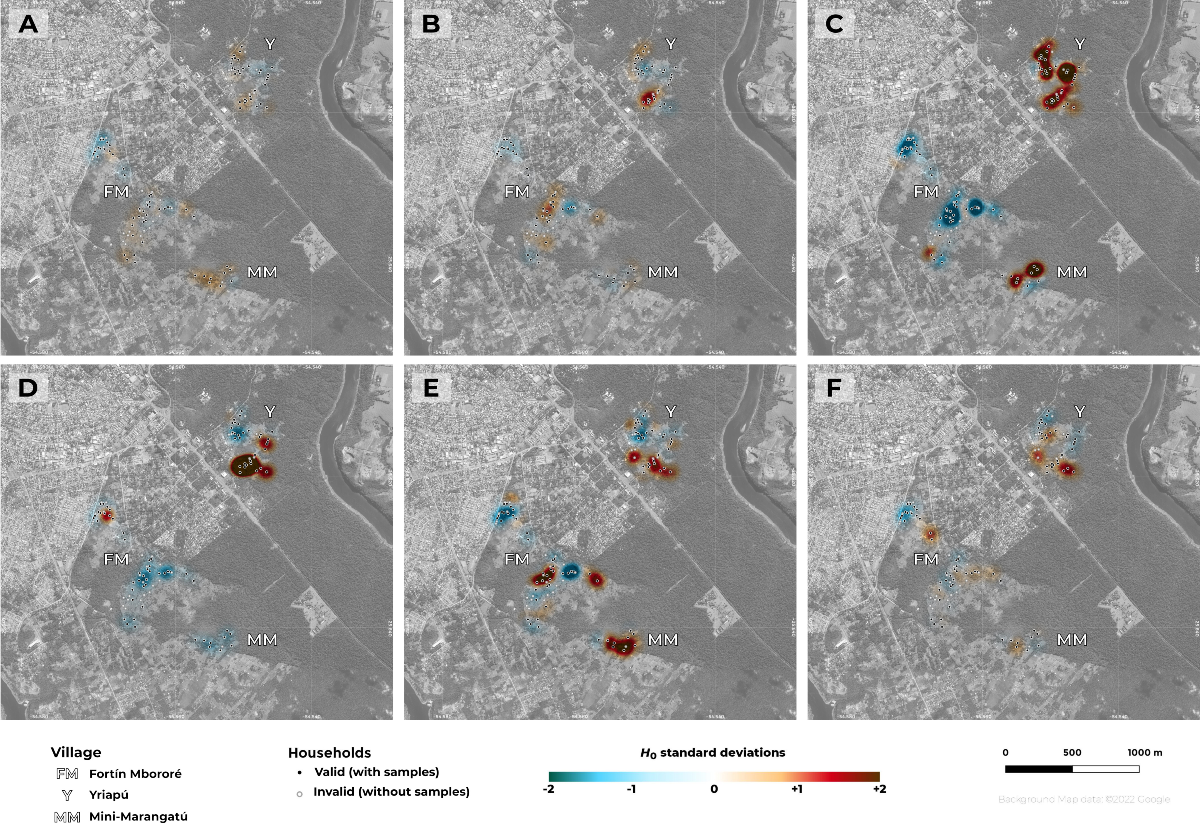
Spatial distribution obtained using the kernel density estimation (KDE) technique for (A) hookworm infection, (B) hookworm intensity, (C) Strongyloides stercoralis infection, (D) Ascaris lumbricoides infection, (E) households with dirt floors where inhabitants walk barefoot, and (F) households with overcrowding. Map created using QGIS with background imagery from Google Maps via the QuickMapServices plugin (version 0.19.29). Copyright 2021 Google. Map data 2021 Google.

The KDE technique was used to analyze the spatial distribution of STH in the study area and observe differences between the villages. As shown in Figure 3A, the distribution of hookworm-positive individuals was not entirely homogenous, and the differences in the SD were not pronounced and were present throughout the entire study area. There was a slight concentration of cases in Mini-Marangatú, followed by Yriapú. Some differences were observed within Fortin Mbororé, where households closer to the urbanized area of Puerto Iguazú (northwest) had lower values than those farther away (southwest).

With respect to the intensity of hookworm infection (Figure 3B), positive cases from Yriapú were mostly of light intensity, while higher-intensity cases were clustered only in the southern area of Yriapú. Although the prevalence of other STH was lower than that of hookworm, the KDE analysis also showed a heterogeneous distribution, with a marked difference in the SD of *S. stercoralis*, showing a high concentration of positive cases in Yriapú and Mini-Marangatú compared to Fortin Mbororé (Figure 3C). *Ascaris lumbricoides* infections were detected mostly in Yriapú village (Figure 3D).

Figure 3E shows the distribution of households with dirt floors and individuals who usually walk barefoot. Given that many houses in Fortin Mbororé had cement floors, the difference in the SD was more evident in Mini-Marangatú and southwestern Fortin Mbororé compared to Yriapú. The distribution of households with overcrowding (Figure 3F) was homogeneous in the study area, although there was a clustering in Yriapú.

The multivariate model used to identify the factors most related to the distribution of hookworm in the study area selected the following predictors: mean VHI, SD of VHI, and ENDISI and households with dirt floors and individuals who walk barefoot. In this model, only the environmental variables were significantly associated *(P*<.001) with the presence of hookworm infection, which explained 56% (248/342) of the variability observed in the distribution of cases in the study area (*F*_108_=34.75, *P*<.001). The predictors for hookworm intensity selected by the multivariate model were the distribution of dirt floor households with individuals that walked barefoot and households with overcrowding. Both variables showed a significant association (*P*<.001) with the intensity of hookworm infection and predicted 45% (153/342) of the variability observed in the distribution of the cases.

## Discussion

### Principal Findings

This study uncovers factors associated with the presence and intensity of STH infection, especially hookworm, in rural areas of Puerto Iguazú. The high prevalence observed coincides with previous studies in other rural areas of the region [5,15,42,43], as well as a higher prevalence in the infant population [3,5]. Hookworm prevalence was above 70% in all villages (114/169 in Fortín Mbororé, 92/125 in Yriapú, and 42/48 in Mini-Marangatú), with the highest prevalence observed in Mini-Marangatú, located in the easternmost area.

In the multivariate analysis for hookworm infection, barefoot walking and overcrowding were implicated in an increase in the intensity of infection, while the type of household floor material was associated with an increase in transmission. These results coincide with those already observed in other studies [12,44,45]. Although other studies indicate that hygienic conditions, water, type of latrine, and level of education are related to a higher hookworm prevalence [16,19,46-48], in this study, these factors did not show statistical significance. These different results may be due to the uniformity of these variables throughout the different villages since most of the households shared the same characteristics. Moreover, although the villages were independent population centers managed by their own community leader or cacique, they were relatively close to each other, thus sharing similar environmental and economic conditions.

Since hygienic conditions have been shown to greatly influence transmission through the fecal-oral route [19], as in the case of *A. lumbricoides*, the proximity to the Mbocay stream in the villages of Fortin Mbororé (eastern zone) and Mini-Marangatú could have generated different *A. lumbricoides* infection rates between the villages. In addition, the high density of construction observed in the western area of Fortin Mbororé, through the ENDISI, may have affected the transmission of this parasite, given that low humidity can hinder egg embryonation [9].

With respect to environmental factors and their predictors, previous studies have highlighted the role of humidity, temperature, and soil type in the transmission of STH [16,45,48,49]. Higher humidity and temperature have been shown to be favorable conditions for the survival of heterogonic stages of *S. stercoralis* [50,51]. A joint analysis of the TPI and TWI allowed us to determine that the village of Yriapú is situated in a depressed area with higher humidity accumulation, which could be a factor behind the high prevalence of *S. stercoralis* observed in this village. Available studies also show the association of humidity and vegetation indices in the prevalence of *S. stercoralis* [52].

To analyze the effect of vegetation on the transmission of STH, the VHI, which was developed specifically for this study, helped identify areas with more or less vegetation with respect to each surrounding household. The algorithm is similar to the TPI; however, instead of elevation, the data source is a vegetational index (in this case, SAVI). High values of this index around households were associated with increased hookworm infection, which indicates the importance not only of the vigorousness of vegetation but also its distribution pattern in the landscape of the study area. High values of this index around the Mini-Marangatú households coincided with the highest prevalence of hookworm in this area. These vegetation and humidity indices, which demonstrate the influence of vegetation on hookworm distribution, coincided with the risk of hookworm infection in areas of VHI values reported in other studies [44,49,53].

Previous studies suggested that infection is more probable in households surrounded by bare soil, among other factors [52,54-56]. Fortin Mbororé has bare soil surrounding its houses, especially in the western area located close to the urbanized area of the city. Although bare soil is an environmental factor that has been shown to be associated with STH infections, this area had lower hookworm infection rates.

The ENDISI aids in detecting impervious surfaces, particularly the amount of water stored. The survival of hookworm larvae depends on the soil’s water-retaining properties. When the soil dries out, the water is restricted to the thin film around individual soil particles, and the infective larval stage remains quiescent in the moisture film until it makes contact with its host [57]. The lower hookworm infection rates observed in Fortin Mbororé may be because this area is highly influenced by the ENDISI since urban development and infrastructure density modify soil permeability and moisture. This can have a negative effect on the persistence and survival of this type of parasite, which requires humidity to survive and develop its larval stages [51,58].

In general, villages sharing similar living conditions and environmental characteristics contributed to the high infection rates observed. This study demonstrated that living conditions play a role in the intensity of hookworm infection, and environmental variables are significantly associated with its presence. However, the specific differences observed in certain areas aid in elucidating how human development and social and sanitary conditions may influence lower infection rates among individuals and villages located in endemic areas. Evidence from previous studies and the results obtained herein show that environmental factors such as temperature, vegetation, and humidity play a role in the presence and maintenance of STHs in the soil. Therefore, environmental changes caused by climate change could modify the distribution of these parasites, although deforestation, bare soil, high temperature, and lack of humidity could restrict their presence.

The limitations of this study include the sensitivity of the techniques used to detect STHs since low burdens of infection could be missed. Additionally, in this area, hookworm was the most prevalent STH; therefore, studies conducted in areas with a greater presence of other species of the group would be beneficial. Further studies in more heterogeneous communities with similar environmental characteristics could help aid our understanding of the socioeconomic and building characteristics that determine the presence and intensity of STH infections.

### Conclusion

This study, conducted in an endemic area for STHs, especially hookworms, reinforces the importance of the environment in the establishment of this group of parasites, which require passage through the soil for their development. Additionally, we observed that living conditions, like walking barefoot, having dirt floors, or overcrowding, are associated with the intensity of hookworm infection. Given that environmental variables cannot be changed, it is important to work on those aspects that can be modified, such as the characteristics of the house, the availability of water and sanitation, and periodic deworming as suggested by the World Health Organization deworming guidelines [59].

## Appendix

Multimedia Appendix 1 Environmental index formulas.

Multimedia Appendix 2 Report of the statistical models used.

## Abbreviations

BSI: Bare Soil Index
DEM: digital elevation model
ENDISI: Enhanced Normalized Difference Impervious Surfaces Index
EVI: Enhanced Vegetation Index
GIS: geographic information system
IP: intestinal parasite
KDE: kernel density estimation
NDVI: Normalized Difference Vegetation Index
NTD: neglected tropical disease
RS: remote sensing
SAVI: Soil-Adjusted Vegetation Index
STH: soil-transmitted helminth
TPI: Topographic Position Index
TWI: Topographic Wetness Index
VHI: Vegetation Heterogeneity Index
WHA: World Health Assembly

## Data Availability

Data supporting the conclusions of this article are included within the article. The datasets used and/or analyzed during this study are available from the corresponding author upon reasonable request.
