# Supplementary material for: The Relationship Between Soil-Transmitted Helminth Infections and Environmental Factors in Puerto Iguazú, Argentina: Cross-Sectional Study
Source: JMIR Public Health Surveill. 2023 Nov 7;9:e41568. doi: 10.2196/41568 (PMC10664009; doi:10.2196/41568)
Supplement: Multimedia Appendix 1 [file publichealth_v9i1e41568_app1.docx]

**Multimedia Appendix 1. Environmental index formulas.**

**Topographic Position Index (TPI)**

$$TPI=Z_{0}-\frac{\sum_{1-n} Z_{n}}{n}$$

Z_0_ = elevation of the model point under evaluation

Z_n_ = elevation of grid within the local window

n = the total number of surrounding points employed in the evaluation

**Vegetation Heterogeneity Index (VHI)**

$$VHI=V_{0}-\frac{\sum_{1-n} V_{n}}{n}$$

V_0_ = Vegetation index value of the model point under evaluation

V_n_ = Vegetation index value of grid within the local window

n = the total number of surrounding points employed in the evaluation

**Topographic wetness index (TWI)**

$$TWI=\frac{\alpha}{tanb}$$

α = local upslope area draining through a certain point per unit contour length

tan b = local slope in radians.

The topographic wetness index is unitless.

**Soil Adjusted Vegetation Index (SAVI)**

$$SAVI=\frac{NIR-R}{NIR+R+L}*1+L$$

NIR = reflectance value of the Near Infrared band

R = reflectance value of the Red band

L = soil brightness correction factor. The value of L varies by the amount or cover of green vegetation: in very high vegetation regions, L=0; and in areas with no green vegetation, L=1. Generally, an L=0.5 works well in most situations and is the default value used. When L=0, then SAVI = NDVI.

**Bare Soil Index (BSI)** [https://ieeexplore.ieee.org/document/1370429]

$$BSI=\frac{\left( R+SWIR \right)-\left( NIR+B \right)}{\left( R+SWIR \right)+\left( NIR+B \right)}$$

R = reflectance value of the Red band

B = reflectance value of the Blue band

SWIR = reflectance value of the Shortwave Infrared band

NIR = reflectance value of the Near Infrared band

**Enhanced Normalized Difference Impervious Surfaces Index (ENDISI)**

$$ENDISI=\frac{B-\alpha*\left( \frac{{SWIR}_{1}}{{SWIR}_{2}}+{MNDWI}^{2} \right)}{B+\alpha*\left( \frac{{SWIR}_{1}}{{SWIR}_{2}}+{MNDWI}^{2} \right)}$$

$$\alpha=\frac{2*B_{mean}}{\left( \frac{{SWIR}_{1}}{{SWIR}_{2}} \right)_{mean}+{MNDWI}_{mean}^{2}}$$

$$MNDWI=\frac{G-{SWIR}_{1}}{G+{SWIR}_{1}}$$

B = reflectance value of the Blue band

G = reflectance value of the Green band

SWIR_1_ = reflectance value of the Shortwave Infrared 1 band

SWIR_2_ = reflectance value of the Shortwave Infrared 2 band

The subscript “Mean” is the mean value of the image.
