# Supplementary material for: The Relationship Between Soil-Transmitted Helminth Infections and Environmental Factors in Puerto Iguazú, Argentina: Cross-Sectional Study
Source: JMIR Public Health Surveill. 2023 Nov 7;9:e41568. doi: 10.2196/41568 (PMC10664009; doi:10.2196/41568)
Supplement: Multimedia Appendix 2 [file publichealth_v9i1e41568_app2.docx]

**Multimedia Appendix 2. Report of the statistical models used.**

**A. Distribution of hookworm positive individuals**

**1. Full Model**

The full multilinear regression model was fitted to explore the relationship between the response variable U_pos_ (distribution of hookworm positive individuals) and several candidate predictors. The model equation is given as:

U_pos_ = 0.946017 - 1.496673 * SAVI - 1.618511 * VHI + 3.053108 * VHIσ - 0.000636 * TWI - 5.127594 * ENDISI - 2.622317 * BSI + 0.056738 * BFDF - 0.069859 * PpR

Coefficients:

|  | Estimate | Std. Error | t value | Pr(>\|t\|) |
| --- | --- | --- | --- | --- |
| (Intercept) | 0.946017 | 0.966848 | 0.978 | 0.3301 |
| SAVI | -1.496673 | 1.167171 | -1.282 | 0.2026 |
| VHI | -1.618511 | 1.062754 | -1.523 | 0.1308 |
| VHIσ | 3.053108 | 1.217828 | 2.507 | 0.0137 * |
| TWI | -0.000636 | 0.010840 | -0.059 | 0.9533 |
| ENDISI | -5.127594 | 0.903960 | -5.672 | 1.28e-07 *** |
| BSI | -2.622317 | 2.092168 | -1.253 | 0.2129 |
| BFDF | 0.056738 | 0.028251 | 2.008 | 0.0472 * |
| PpR | -0.069859 | 0.053992 | -1.294 | 0.1986 |

Significancy codes: 0 ‘***’ 0.001 ‘**’ 0.01 ‘*’ 0.05 ‘.’ 0.1 ‘ ’ 1

The results indicate that only the predictor ENDISI had a highly significant effect on U_pos_ (p < 1.28e-07). Other predictors such as VHIσ and BFDF also showed significant relationships (p < 0.05).

Model Statistics:

Residual standard error: 0.3038

Multiple R-squared: 0.5767

Adjusted R-squared: 0.5441

F-statistic: 17.71 on 8 and 104 DF

p-value: < 2.2e-16

**2. AIC Stepwise Variable Selection**

AIC stepwise variable selection was applied to identify the most significant predictors for the reduced model. The algorithm suggested retaining the following predictors: VHI, VHIσ and ENDISI.

These variables were found to have the greatest impact on the response variable U_pos_.

**3. Reduced Model**

The reduced multilinear regression model was fitted using the selected predictors from the AIC stepwise process. The model equation is given as:

U_pos_ = -0.39445 - 2.31023 * VHI + 3.13706 * VHIσ - 4.03425 * ENDISI

Coefficients:

|  | Estimate | Std. Error | t value | Pr(>\|t\|) |
| --- | --- | --- | --- | --- |
| (Intercept) | -0.39445 | 0.17632 | -2.237 | 0.02734 * |
| VHI | -2.31023 | 0.76057 | -3.037 | 0.00299 ** |
| VHIσ | 3.13706 | 1.07241 | 2.925 | 0.00420 ** |
| ENDISI | -4.03425 | 0.50338 | -8.014 | 1.4e-12 *** |

Significancy codes: 0 ‘***’ 0.001 ‘**’ 0.01 ‘*’ 0.05 ‘.’ 0.1 ‘ ’ 1

The results show that all selected predictors have significant effects on U_pos_, as their p-values are less than 0.05.

Model Statistics:

Residual standard error: 0.303

Multiple R-squared: 0.5627

Adjusted R-squared: 0.5465

F-statistic: 34.75 on 3 and 108 DF

p-value: < 2.2e-16

**4. Coefficient Confidence Intervals**

The 95% confidence intervals for the coefficients of the reduced model are as follows:

(Intercept): [-0.74395, -0.04494]

SAVI_TPI_2hamean: [-3.81782, -0.80264]

SAVI_TPI_2hastdev: [1.01136, 5.26276]

Dens_Constr_2hamean: [-5.03203, -3.03646]

These intervals indicate the range of plausible values for each predictor coefficient.

**B. Intentisity of hookworm infection**

**1. Full Model**

The full multilinear regression model was fitted to explore the relationship between the response variable U_int_ (intensity of hookworm infection) and several candidate predictors. The model equation is given as:

U_int_ = -0.92734 + 1.32154 * SAVI - 2.31871 * VHI + 0.20155 * VHIσ - 0.02246 * TWI - 0.96314 * ENDISI + 3.88593 * BSI + 0.33735 * BFDF - 0.28546 * PpR

Coefficients:

|  | Estimate | Std. Error | t value | Pr(>\|t\|) |
| --- | --- | --- | --- | --- |
| (Intercept) | -0.92734 | 1.42362 | -0.651 | 0.516231 |
| SAVI | 1.32154 | 1.71859 | 0.769 | 0.443653 |
| VHI | -2.31871 | 1.56484 | -1.482 | 0.141429 |
| VHIσ | 0.20155 | 1.79318 | 0.112 | 0.910723 |
| TWI | -0.02246 | 0.01596 | -1.407 | 0.162313 |
| ENDISI | -0.96314 | 1.33102 | -0.724 | 0.470933 |
| BSI | 3.88593 | 3.08059 | 1.261 | 0.209978 |
| BFDF | 0.33735 | 0.04160 | 8.110 | 1.06e-12 *** |
| PpR | -0.28546 | 0.07950 | -3.591 | 0.000505 *** |

Significancy codes: 0 ‘***’ 0.001 ‘**’ 0.01 ‘*’ 0.05 ‘.’ 0.1 ‘ ’ 1

The results indicate that the predictors BFDF and PpR had highly significant effects on U_int_ (p < 1.06e-12 and p < 0.000505, respectively). Other predictors, such as VHI, BSI, and SAVI, also showed significant relationships (p < 0.05).

Model Statistics:

Residual standard error: 0.4473

Multiple R-squared: 0.5167

Adjusted R-squared: 0.4795

F-statistic: 13.9 on 8 and 104 DF

p-value: 1.449e-13

**2. AIC Stepwise Variable Selection**

AIC stepwise variable selection was applied to identify the most significant predictors for the reduced model. The algorithm suggested retaining the following predictors: BFDF, PpR.

These variables were found to have the greatest impact on the response variable U_int_.

**3. Reduced Model**

The reduced multilinear regression model was fitted using the selected predictors from the AIC stepwise process. The model equation is given as:

U_int_ = 0.05234 + 0.35294 * BFDF - 0.20609 * PpR

Coefficients:

|  | Estimate | Std. Error | t value | Pr(>\|t\|) |
| --- | --- | --- | --- | --- |
| (Intercept) | 0.05234 | 0.04427 | 1.182 | 0.23965 |
| BFDF | 0.35294 | 0.03672 | 9.611 | 3.05e-16 *** |
| Pers_cuart | -0.20609 | 0.06904 | -2.985 | 0.00349 ** |

Significancy codes: 0 ‘***’ 0.001 ‘**’ 0.01 ‘*’ 0.05 ‘.’ 0.1 ‘ ’ 1

The results show that all selected predictors have significant effects on U_int_, as their p-values are less than 0.05.

Model Statistics:

Residual standard error: 0.4612

Multiple R-squared: 0.4566

Adjusted R-squared: 0.4467

F-statistic: 46.21 on 2 and 110 DF

p-value: 2.712e-15

**4. Coefficient Confidence Intervals**

The 95% confidence intervals for the coefficients of the reduced model are as follows:

(Intercept): [-0.29053, 0.51181]

BFDF: [0.28702, 0.44048]

PpP: [-0.36347, -0.09373]

These intervals indicate the range of plausible values for each predictor coefficient.
